# Presence of papillomavirus sequences in condylomatous lesions of the mamillae and in invasive carcinoma of the breast

**DOI:** 10.1186/bcr940

**Published:** 2004-10-22

**Authors:** Ethel-Michele de Villiers, Robert E Sandstrom, Harald zur Hausen, Charles E Buck

**Affiliations:** 1 Division for the Characterization of Tumorviruses, Deutsches Krebsforschungszentrum, Heidelberg, Germany; 2 Lower Columbia Pathologists, Longview, Washington, USA

**Keywords:** areolar tissue, breast carcinoma, papillomavirus

## Abstract

**Background:**

Viruses including Epstein–Barr virus (EBV), a human equivalent of murine mammary tumour virus (MMTV) and human papillomavirus (HPV) have been implicated in the aetiology of human breast cancer. We report the presence of HPV DNA sequences in areolar tissue and tumour tissue samples from female patients with breast carcinoma. The presence of virus in the areolar–nipple complex suggests to us a potential pathogenic mechanism.

**Methods:**

Polymerase chain reaction (PCR) was undertaken to amplify HPV types in areolar and tumour tissue from breast cancer cases. *In situ *hybridisation supported the PCR findings and localised the virus in nipple, areolar and tumour tissue.

**Results:**

Papillomavirus DNA was present in 25 of 29 samples of breast carcinoma and in 20 of 29 samples from the corresponding mamilla. The most prevalent type in both carcinomas and nipples was HPV 11, followed by HPV 6. Other types detected were HPV 16, 23, 27 and 57 (nipples and carcinomas), HPV 20, 21, 32, 37, 38, 66 and GA3-1 (nipples only) and HPV 3, 15, 24, 87 and DL473 (carcinomas only). Multiple types were demonstrated in seven carcinomas and ten nipple samples.

**Conclusions:**

The data demonstrate the occurrence of HPV in nipple and areolar tissues in patients with breast carcinoma. The authors postulate a retrograde ductular pattern of viral spread that may have pathogenic significance.

## Introduction

Breast cancer is the most frequently diagnosed cancer among women in the USA, and the incidence of breast carcinoma has increased by more than 40% over 25 years [1]. The aetiology of breast cancer remains unknown. Many risk factors have been associated with the pathogenesis of this disease, including family history, hormones, cigarette smoking and alcohol consumption [2-7]. Hormones, cigarette smoking and family history have also been demonstrated to enhance infections with papillomaviruses, mainly the high-risk human papillomavirus (HPV) types involved in the aetiology of cervical carcinoma [8].

Viral infection as an aetiological factor has been addressed in other studies. The data obtained from studies investigating the presence of viral sequences in breast cancer biopsies and cell lines have been controversial. A role for herpes viruses, and specifically Epstein–Barr virus (EBV), in the aetiology of this cancer has been questioned [9,10]. A human equivalent of the murine mammary tumour virus (MMTV) has been described in breast tumours, as well as in breast cancer cell lines [11]. These results have been questioned and negated by others (R Schmidt and H zur Hausen, unpublished results) [12]. Immortalisation of normal breast epithelial cells by the HPV types HPV 16 and HPV 18 [13] has been used to study the functions of the viral early genes E6 and E7 in different cellular pathways [14-18]. However, the presence of HPV in malignant tumours of the breast has been controversial. Several studies reported positive results [19-25], whereas others reported negative results [26-28]. In the present study we report the presence of HPV DNA in biopsy samples from mammary carcinomas, as well as these viral sequences in the tissue of the corresponding mamilla.

## Materials and methods

### Samples and DNA extraction

Samples from breast tumour tissue for the HPV analysis were supplied by two of the authors (RES and CEB). The 29 cases used for the retrospective study and demonstration of papillomavirus sequences were selected from routine mastectomy specimens undertaken in a course of treatment for carcinoma of the breast. Case selection was based on the availability of adequate nipple and tumour tissue from the same patient and the presence of histological features suggestive of HPV infection in nipple and areolar sections. The patients, all female, ranged in age from 30 to 88 years, with an average age of 61.3 years. Twelve tumours arose in the right breast and 17 in the left. Five tumours were subareolar and 24 were located at a distance from the nipple and areola. Tumours ranged in size from 6 mm to 15 cm. One patient received cytoxan chemotherapy before surgery. In all other cases primary treatment was surgical as represented by the specimen. One patient had undergone contralateral mastectomy ten years earlier for a localised breast cancer, which was judged clinically and pathologically to represent a separate primary tumour. Among the 29 patients there were three with remote histories of malignancy without evidence of recurrence (one cutaneous malignant melanoma, one renal cell carcinoma and one soft-tissue malignancy). One patient had undergone hysterectomy for high-grade cervical dysplasia. There were no patients with documented cervical carcinoma. No patients were immune-impaired or immunosuppressed.

Formalin-fixed paraffin-embedded samples from both the nipple (*n *= 29) and the carcinoma of the breast (*n *= 29) from the same patient were sectioned, and the total DNA was extracted from each sample. Great care was taken during sectioning to avoid any contamination between samples. Two different individuals sectioned the blocks in several small random groups at different time intervals over a period of one week. The microtome was cleaned thoroughly and exposed to ultraviolet between samples; in addition, new blades were used for each sample. Deparaffinisation was performed by rotation overnight in 1 ml of xylene, followed by centrifugation and subsequent removal of the supernatant. This step was repeated twice (each for one hour) with fresh xylene. The xylene was in turn removed by rotation in 1 ml of 100% ethanol for one hour followed by centrifugation and subsequent replacement of the supernatant with 90% ethanol for 45 min, and repeated by steps of 80% ethanol for 45 min and 70% ethanol for 45 min. Samples were freeze-dried, and total cellular DNA was extracted as described previously [29]. The DNA was digested with Proteinase K and subjected to extraction with phenol followed by extraction with chloroform/isoamyl alcohol. The extracted DNA was precipitated with ethanol and the pellet was resuspended in 10 mM Tris/HCl, pH 8.0.

### PCR analysis and cloning

DNA (50–100 μg) of each sample was amplified by polymerase chain reaction (PCR). The quality of the DNA obtained from the fixed samples was controlled by amplification with primers to detect the β-actin gene [30]. The primers RS42 and KM29 were initially used to amplify the 536-base-pair fragment of the β-actin gene. If this size of fragment could not be amplified, the primers PC03 and PC04 were used to amplify the 110-base-pair (bp) fragment of the β-actin gene. Papillomavirus sequences were amplified by three different methods, each targeting highly conserved regions within the L1 open reading frame of papillomaviruses. These included the GP5^+^/GP6^+ ^primers [31], the CP primers using modified conditions as previously described [32] and the FAP primers [33]. All three primer combinations are routinely used on individual samples tested in our laboratory. We initially modified the PCR conditions for each described method to include the amplification of the largest number of individual HPV types possible. Cloned DNA of each known HPV type was used to optimise these conditions. Amplification with the GP primers was performed in 2 mM MgCl_2 _using 40 cycles with an annealing temperature of 40°C. The expected size of the amplicon was 140–150 bp. The expected size with the FAP primers is 480 bp after 45 cycles in 3.5 mM MgCl_2 _and an annealing temperature of 50°C. The nested amplification approach with the CP primers results in an amplicon size of 370–400 bp. The initial amplification was performed in 2 mM MgCl_2 _and 40 cycles at 50°C annealing temperature, after which an aliquot was re-amplified with nested primers but with the same PCR conditions. All amplicons were eluted after gel electrophoresis, purified and cloned. At least ten fragments of each amplicon were sequenced. Sequencing was performed with either the Sequenase 2.0 DNA Sequencing Kit (USB, Cleveland, OH) or an ABI Model Sequencer with Big Dye Terminator chemistry (Perkin Elmer Applied Biosystems Division, Dreieich, Germany). All precautions were taken to avoid contamination before or during extraction of the DNA from the embedded formalin-fixed samples. After initial results had been obtained, a second sectioning was performed on the same embedded samples and DNA was again extracted to ensure that no contamination had taken place during the first round of experiments. This second round of analyses was performed in different rooms and by different individuals.

### Sequence analysis

Sequences were compared with all available data banks with the aid of the Husar software package (Deutsches Krebsforschungszentrum).

### *In situ *hybridisation

*In situ *hybridisation was performed as described by Kawase and colleagues [34]. Papillomavirus probes were digoxigenin-labelled by PCR in accordance with the manufacturer's instructions (PCR DIG Probe Synthesis Kit, catalogue no. 1636090; Roche, Mannheim, Germany). The FAP primers were used to label HPV 6 and HPV 11 DNA and the CP primers to label HPV 16 DNA. Denaturation was performed in a Biozym cycler for 5 min at 95°C followed by hybridisation overnight at 37°C under stringent conditions as described previously [35]. This was followed by washing with solutions in accordance with instructions in the Genpoint Kit (catalogue no. K0620; Dako, Hamburg, Germany). Anti-digoxigenin-AP antibodies (anti-digoxigenin-AP, Fab fragments, catalogue no. 1093274; Roche), blocking reagent (catalogue no. 1096176; Roche) and Nitro Blue Tetrazolium/5-bromo-4-chloroindol-3-yl phosphate (catalogue no. 1681451; Roche) were applied for visualisation of the signals. Sections from each sample were used for *in situ *hybridisation. Reading of the samples was blinded and performed by one of us (HzH).

## Results

**Figure 1:**
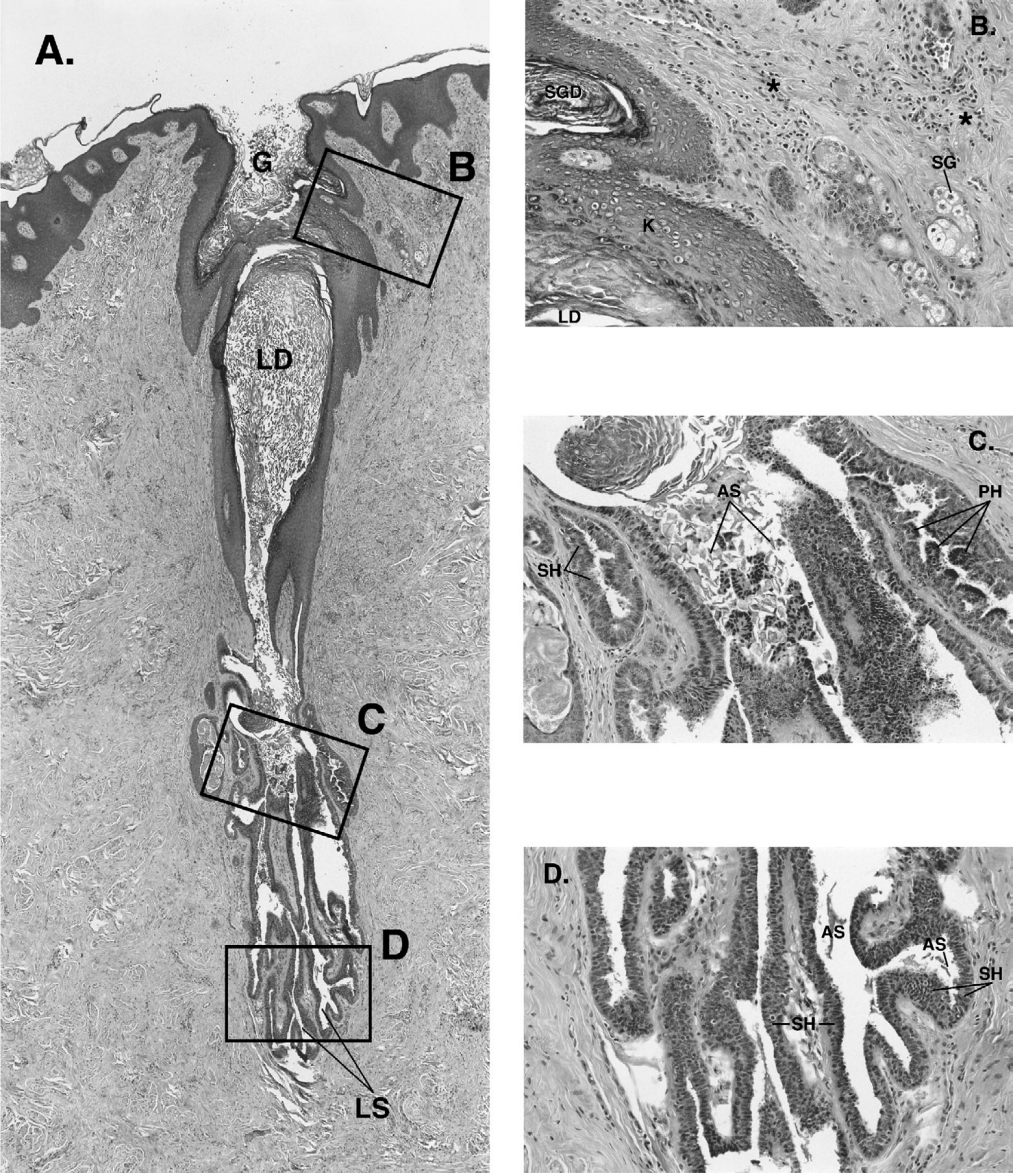
Histological features of the carcinomatous breast (magnification × 100). Detailed histology of **(a) **is seen in **(b–d)**. AS, anucleate squames; asterisks, interstitial lymphocytic infiltrates; G, galactophore; K, keratin; LS, lactiferous sinus; LD, lactiferous duct; SG, sebaceous gland (tangenial cut); SGD, sweat gland duct; SH, simple epithelial hyperplasia; PH, papillary epithelial hyperplasia.

Nipple specimens were reviewed and selected by two of us (CEB, RES) for histological features consistent with human papillomavirus infections. Features identified included epithelial hyperplasia, hypergranulosis, parakeratosis, and distorted nuclear shape associated with nuclear clearing and pyknosis suggestive of koilocytosis. In addition, squamous metaplasia of lactiferous ducts and shedding of metaplastic cellular elements into lactiferous ducts and sinuses were noted (Fig. 1). In addition to the 29 invasive carcinomas, ductal carcinoma *in situ *was identified in lactiferous ducts in four samples and small benign papillomas were present in three samples. Most invasive carcinoma samples (18 of 29) displayed a duct cell carcinoma pattern (Table 1). Other types included medullary carcinoma (five cases), tubular carcinoma (three cases), lobular carcinoma (two cases) and mucinous carcinoma (one case). DNA extracted from the samples taken from the nipple and the corresponding carcinoma of each breast (29 pairs) was analysed by PCR for the presence of papillomavirus sequences. The quality of the DNA was controlled by amplification of the β-actin gene. The 536-base-pair fragment of this gene was amplified in 47 of 58 samples. The 110-base-pair fragment was amplified in the remaining 11 samples (Table 2). Intact papillomavirus particles are resistant to degeneration. Extraction of DNA from fixed tissue can harbour intact viral genomes even under conditions in which the cellular DNA has been degraded. The three different methods used for amplification of the papillomavirus DNA were chosen to ensure amplification of all known papillomavirus types, as well as putative new types. All three primer combinations (GP, CP and FAP) are routinely used on individual samples tested in our laboratory. We initially modified the PCR conditions for each described method to include the amplification of the largest number of individual HPV types possible. Cloned genomic DNA of each known HPV type was used to optimise these conditions. The GP primers have been designed to amplify the majority of papillomavirus types associated with mucosal lesions. The FAP primers, in contrast, were initially designed to amplify the majority of papillomavirus types associated with cutaneous lesions. The amplicon size described is 480 bp, but in our hands smaller fragments identified as papillomavirus sequences (after cloning and sequencing) were also generated, depending on the HPV type. The CP primers were originally designed to amplify all the epidermodysplasia verruciformis-associated HPV types, but under modified conditions a large spectrum of mucosal types are amplified as well. Combining the three methods with the modified PCR conditions allows us to amplify all known HPV types with more or less equal efficiency. The amplicons generated, using any of the described methods, often constitute cellular sequences depending on the viral load of the sample.

Normal breast and areolar tissue was not available for use as control samples. The described methods of testing have been used routinely in our laboratory over several years in the analyses of tissues from a large variety of organs. We have analysed large numbers of samples from benign and malignant tumours of the oesophagus, head and neck and skin, as well as normal skin tissue for the presence of papillomavirus DNA with identical methods. HPV positivity varied depending on the type of tumour and normal tissue [29,36-38] (E-MdV, unpublished data). The present study describes the first series of tissue of the breast analysed in our laboratory for the presence of papillomavirus DNA. Sequence analyses showed that 25 (86%) of the breast carcinoma samples and 20 (69%) of the samples from the mamilla harboured papillomavirus sequences. The results for each sample using each of the primer combinations are presented in Table 2. Only one pair of samples were both negative for HPV DNA. HPV DNA was present in 17 (59%) pairs of samples, namely nipple as well as carcinoma, whereas papillomavirus DNA was detected only in the carcinomatous tissue in eight cases (28%) and only in the nipple in three cases (10%) (Table 3). HPV 11 DNA was present in both carcinoma and nipple samples in seven cases, HPV 6 in one case and HPV 57 in two cases. The most prevalent HPV types were HPV 11 (19 carcinomas and 8 nipples) followed by HPV 6 (7 carcinomas and 6 nipples) (Table 4). Other HPV types detected were HPV 16 (2 carcinomas and 3 nipples), HPV 57 (2 carcinomas and 3 nipples), HPV 27 (2 carcinomas and 4 nipples), HPV 66 (2 nipples), candHPV 87 (4 carcinomas), HPV 37 (4 nipples) and the putative new HPV type DL250 (HPV 9-related) in two carcinomas. HPV types detected in single samples of the nipple were HPV 20, 21, 23, 32, 38 and GA3-1 (HPV 8-related) and HPV 3, 15, 23, 24 and DL473 (HPV 15-related) in carcinoma samples. Multiple HPV types were demonstrated in eight carcinomas (24%) and ten nipple (34.5%) samples.

**Figure 2:**
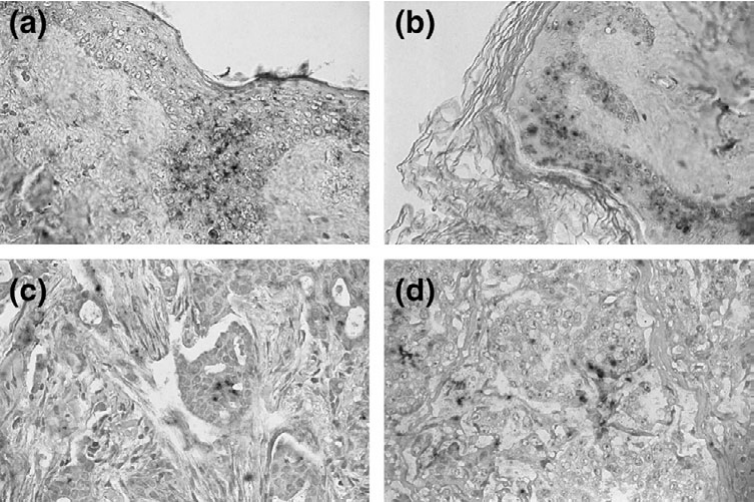
Papillomavirus sequences detected by *in situ *hybridisation. **(a) **Case 10012, in the nipple epithelium (magnification × 40). **(b) **Case 10041, in the nipple epithelium (magnification × 40). **(c) **Case 10012, in tumour tissue (magnification × 100). **(d) **Case 10014, in tumour tissue (magnification × 100).

*In situ *hybridisation was performed on all samples that had previously been positive by PCR amplification for HPV 6, HPV 11 or HPV 16. Positive signals were clearly distinguishable from the surrounding negative tissue (Fig. 2). All carcinoma samples that had been negative for one of these three HPV types by PCR were also subjected to *in situ *hybridisation with each of the respective labelled HPV probes. No positive hybridisation signal was observed in any of these samples. *In situ *hybridisation supported the presence of HPV 6, 11 and 16 in nipple and areolar samples and in all tumour tissue in which these type-specific HPV sequences were detected by PCR analysis.

## Discussion

The ubiquitous distribution of papillomavirus infections of the skin and genital and oral mucosa has been documented [39-41]. Infection with specific papillomavirus types has been shown to be a necessary but not sufficient cause in the pathogenesis of malignant genital tumours. The malignant tumours generally develop after long latency periods during which additional cellular modifications occur within the infected cell [8]. The E6 and E7 genes of the most prevalent high-risk HPV types, HPV 16 and HPV 18, modulate cellular pathways, thereby regulating proliferation and cell survival [8,42]. In contrast, the E6 and E7 proteins of the low-risk types, HPV 6 and HPV 11, do not influence these cellular pathways in the same manner, although they have occasionally been demonstrated in premalignant and malignant tumours. Additional cofactors are probably needed to modulate cellular proteins so as to immortalise and transform the infected cells [8]. The mechanism through which other HPV types, mainly associated with cutaneous lesions, might be involved in the pathogenesis of tumours has received little attention. However, preliminary data indicate that several molecular pathways are probably followed, depending on the HPV type involved [43].

The detection of papillomavirus infections in tissues largely depends on the method used. Described methods vary greatly in their sensitivity and specificity. The HPV types HPV 6, 11, 16, 18, 31, 33, 35, 39, 52 and 58 are frequently associated with genital lesions and are therefore most often targeted for HPV detection. Tests to demonstrate any of the 96 characterised HPV types in tissues require extensive analyses, including the sequencing of cloned amplimers. Most published studies therefore used methods restricted to the detection of specific, single, or combinations or groups of HPV types. The use of type-specific primers may increase the number of positive samples but is biased with regard to the HPV types involved, because other HPV types present cannot be detected.

The present study investigated breast samples by using DNA PCR amplification of two conserved regions within the L1 open reading frame of the papillomavirus genome. Two of the primer combinations used (CP and GP primers) amplify an overlapping region within the L1 open reading frame. By using all three primer combinations to amplify the DNA of a single sample we are able to detect all known HPV types and also putative new HPV types. In addition, the cloning of the amplified product, in combination with sequencing of the cloned inserts, allows us to distinguish individual HPV types present in one sample, including multiple infections in one sample. Studies applying the techniques described here have demonstrated a larger spectrum of papillomavirus types associated with different types of benign and malignant tumours and argue that the historical grouping into 'mucosal' and 'cutaneous' HPV types can no longer be upheld [44]. We are not aware of any study outside our group that has analysed tissues for the presence of infection by any HPV types as extensively as the study described here.

Other investigators have analysed nipple and areolar specimens for the presence of HPV DNA. HPV 6/11 was detected in one of 20 papillomas of the nipple, whereas ten nipple duct adenomas were negative [45] and seven low-risk and high-risk HPV types were not present in 20 cases of Paget's disease of the nipple [46]. Single cases of papillomas of the nipple resembling condylomata acuminata harboured HPV 6 DNA [47] and HPV 41 DNA [48]. Reports on the detection of HPV 16, HPV 18 and/or HPV 33 DNA in breast carcinoma samples range between 11% and 68% positivity [19-25]. The present study detected HPV 16 DNA in 7% of the carcinoma and in 10% of the nipple samples. The only other high-risk HPV type, HPV 66, was present in two samples from the nipple. The reasons for the differences in published reports are unclear, but may be attributed to numbers of samples tested, methodological differences or the demographics of the samples tested. In contrast, several other studies failed to demonstrate HPV sequences in tumours of the breast: all of 15 papillomas and 28 breast carcinoma samples were negative for HPV 6, 16 and 18 DNA [26], no HPV 16 and HPV 18 DNA could be detected in 26 fine needle aspirates and four breast carcinoma biopsies [28], and no HPV DNA was found in 80 samples of breast carcinomas [27]. HPV 16 and HPV 18 oncogenes immortalise normal human mammary cells under *in vitro *conditions [14-18]. It is unclear whether these data truly represent the *in vivo *situation.

The present study detected papillomavirus sequences in 86% (25/29) of the breast carcinoma samples and 69% (20 of 29) of the nipple samples. HPV 6 and HPV 11 infections were most prevalent in our collection of samples. Either of the two HPV types was present in 20 of 29 (69%) of the breast carcinoma biopsies and in 12 of 29 (41%) of the samples of the mamilla. We detected the simultaneous presence of more than one HPV type in about one-third of the samples. HPV types associated with mucosal lesions, and also several associated with skin lesions, were detected. HPV 27 and HPV 57 are closely related HPV types. HPV 27 is very frequently found in cutaneous warts, whereas HPV 57 has been demonstrated in mucosal and cutaneous lesions [49]. Several other HPV types found in the breast samples have historically been associated with the rare hereditary disease epidermodysplasia verruciformis but have more recently been shown in non-melanoma skin cancers and their precursors in both immunosuppressed and immunocompetent patients [44].

The confirmation of PCR-derived evidence of HPV sequences in nipple, areolar and tumour tissue by *in situ *hybridisation in our hands lends substantive support to the conjecture that viral sequences are present and not an artefact in the tissues we have examined. The absence of HPV DNA in several samples, as well as the detection of different HPV types in the carcinoma versus the nipple of the same patient, might be attributed to the viral load at the time of sampling in combination with the sensitivity of the tests performed. The tissue sections also harboured unaffected tissue, leading to the dilution of virus-containing cells. In addition, the amplification of viral DNA by PCR with degenerate or consensus primers will detect replicating viral sequences and is not sensitive enough to detect all individual HPV types with equal sensitivity in a mixed population of cells. The *in situ *hybridisation technique is also less sensitive and will not detect a single viral genome copy per cell.

HPV 6 and HPV 11 are regarded as low-risk HPV types because they have only rarely been demonstrated in premalignant or malignant tissue, and the early genes E6 and E7 do not immortalise primary keratinocytes *in vitro *[50]. HPV 6 DNA has been found in carcinomas [51,52] and giant condylomas (Buschke–Löwenstein tumours) [53,54]. The molecular mechanism by which these low-risk HPV types induce or participate in the transformation of cells has not been resolved. Duplications of the long control region [54-57], minimal sequence dissimilarities [58,59] and integration [60] of HPV 6 and/or HPV 11 genomes have been shown in malignant tissue.

The mere presence of the low-risk HPV 6 and 11 in most of the mamillae and in the concomitant breast carcinoma samples does not prove its role in the aetiology of the disease, although it merits further investigation. Unfortunately, because of the previous fixation of our samples, RNA from these tumours was not available for the detection of viral transcripts as additional support of our results. Relatively few studies have been performed in an attempt to unravel the intracellular mechanisms through which HPV 6 or 11 may immortalise cells, and information is still fractional in comparison with data on the high-risk HPV types. Introduction of the HPV 6 E6 into normal mammary cells leads to immortalisation and reduced levels of the p53 protein [61]. Binding of the HPV 6 E6 protein modulates the function of the cellular proteins Bak [62], Gps2 [63] and Zyxin [64]. Additional *in vitro *studies investigating the influence of hormones, smoke adducts and genetic factors on the interaction of the HPV 6 and 11 proteins with cellular proteins should provide valuable information on a possible role for HPV 6 and 11 in the pathogenesis of breast carcinoma. The data presented in this study also indicate a strong need for epidemiological studies correlating HPV, cigarette smoking, hormone use and family history to substantiate *in vitro *findings.

This study suggests that human papillomavirus may infect the epithelium of the nipple and areola. Human papillomavirus infection may be identified with recognisable histological features comparable to human papillomavirus infection at other sites. Furthermore, this study is consistent with a pathogenic mechanism involving transfer in a retrograde fashion via the nipple, areola, lactiferous ducts and sinuses of the human papillomavirus. If confirmed, the pathogenic mechanism proposed may have significant implications for the prevention and treatment of breast carcinoma and the identification of individuals at risk for carcinoma development. Examination of cellular samples of nipple and areola inclusive of cytological examination and methods for identifying the presence of human papillomavirus might aid early diagnosis and perhaps therapy.

## Conclusions

The data demonstrate the occurrence of HPV in nipple and areolar tissues in patients with breast carcinoma. The presence of papillomavirus DNA in most mamillae and concomitant breast carcinoma samples merits further investigation.

## Competing interests

The author(s) declare that they have no competing interests.

## Abbreviations

EBV = Epstein–Barr virus; HPV = human papillomavirus; MMTV = murine mammary tumour virus; PCR = polymerase chain reaction.

## Figures and Tables

**Table 1:** Samples analysed for HPV sequences

Carcinoma type	Location	Number tested	HPV DNA positive
Invasive ductal	Carcinoma	18	16
	Nipple		11
Medullary	Carcinoma	5	4
	Nipple		3
Tubular	Carcinoma	3	3
	Nipple		3
Lobular	Carcinoma	2	1
	Nipple		2
Mucinous	Carcinoma	1	1
	Nipple		1
Total carcinoma	Carcinoma	29	25 (86%)
	Nipple		20 (69%)

HPV, human papillomavirus.

**Table 2:** Presence of HPV DNA: results obtained after amplification with respective primers

Carcinoma type	Patient number	Location	β-actin		HPV		
			
			KM29/RS42 (536 bp)	PC03/PC04 (110 bp)	GP	CP	FAP
Invasive duct cell	1	Carcinoma	+		HPV11	-	HPV11
		Nipple	+		HPV16	HPV27	HPV11, HPV57
	2	Carcinoma	+		HPV11	-	HPV11
		Nipple	-	+	HPV11	-	HPV6, HPV11
	3	Carcinoma	+		-	-	HPV11
		Carcinoma	+		HPV16	HPV27	-
	4	Carcinoma	+		-	-	HPV11
		Nipple	-	+	-	-	-
	5	Carcinoma	+		-	-	HPV 11
		Nipple	+		-	-	HPV3, HPV11, HPV20, HPV21
	6	Carcinoma	+		HPV11, HPV16	HPV6, HPV27, HPV57	HPV3, HPV6, HPV57
		Nipple	+		-	-	HPV6, HPV11, HPV57
	7	Carcinoma	+		-	-	-
		Nipple	+		-	-	-
	8	Carcinoma	+		-	DL250 (HPV9-related)	-
		Nipple	+		-	-	-
	9	Carcinoma	+		-	DL473 (HPV15-related)	-
		Nipple	+		-	-	-
	10	Carcinoma	+		-	HPV6	-
		Nipple	+		-	-	-
	11	Carcinoma	+		-	HPV23	-
		Nipple	+		-	GA3-1 (HPV8-related)	-
	12	Carcinoma	+		HPV11	-	-
		Nipple	+		-	HPV37	-
	13	Carcinoma	-	+	HPV11	-	HPV11
		Nipple	-	+	-	-	HPV6
	14	Carcinoma	-	+	-	HPV 24	-
		Nipple	+		-	-	-
	15	Carcinoma	+		HPV11	HPV6, DL250	HPV11, HPV87
		Nipple	+		HPV66	-	-
	16	Carcinoma	+		-	-	HPV11
		Nipple	+		-	-	-
	17	Carcinoma	+		-	-	-
		Nipple	-	+	HPV6, HPV66	HPV37	-
	18	Carcinoma	+		HPV11	-	HPV11
		Nipple	+		HPV6	HPV37	-
Medullary	19	Carcinoma	+		HPV11	-	HPV11
		Nipple	-	+	-	-	HPV11
	20	Carcinoma	+		-	-	-
		Nipple	+		HPV11	-	-
	21	Carcinoma	+		HPV11	HPV6	HPV6, HPV87
		Nipple	+		-	HPV23	-
	22	Carcinoma	+		HPV11	HPV6	HPV6, HPV87
		Nipple	+		-	-	-
	23	Carcinoma	+		-	HPV 6	HPV11
		Nipple	+		-	-	-
Tubular	24	Carcinoma	+		-	-	HPV57
		Nipple	-	+	HPV6, HPV57	HPV6, HPV57	HPV6, HPV57
	25	Carcinoma	+		-	-	HPV11
		Nipple	-	+	HPV16, HPV27	-	HPV11
	26	Carcinoma	+		HPV11	HPV15	HPV87
		Nipple	-	+	-	HPV27, HPV37	-
Lobular	27	Carcinoma	+		HPV11, HPV16	HPV27	-
		Nipple	+		-	-	HPV11
	28	Carcinoma	+		-	-	-
		Nipple	+		-	-	-
Mucinous	29	Carcinoma	-	+	-	HPV6	HPV11
		Nipple	+		-	HPV38	-

bp, base pairs; HPV, human papillomavirus.

**Table 3:** Papillomavirus sequences demonstrated

Tumor pairs	Number	HPV types^a^
Carcinoma^+ ^nipple^+^	17	Same type in both samples: 11 (7×)^b^, 6 (1×)^b^, 57 (2×) Different types: 3, 15, 16, 20, 21, 23, 27, 37, 38, 66, 87, DL250, DL473, GA3-1
Carcinoma^- ^nipple^+^	3	6, 11, 32, 37, 66
Carcinoma^+ ^nipple^-^	8	6, 11, 24, 87, DL250, DL473
Carcinoma^- ^nipple^-^	1	

^a^Infection with multiple human papillomavirus (HPV) types was demonstrated in eight carcinoma samples (27.6%) and ten nipple samples (34.5%).

^b^One pair harboured HPV 6, HPV 11 and HPV 57 DNA.

**Table 4:** Types of papillomavirus detected

Carcinoma type	Location	HPV types									
		
		6	11	16	27	37	57	66	87	DL250	Others
Intraductal	Carcinoma	3	11	1	1	-	1	-	1	2	3, 23, 24, DL473
	Nipple	5	4	2	2	3	2	2	-	-	20, 21, GA3-1
Lobular	Carcinoma	-	1	1	1	-	-	-	-	-	-
	Nipple	-	1	-	-	-	-	-	-	-	32
Tubular	Carcinoma	-	2	-	-	-	1	-	1	-	15
	Nipple	1	1	1	2	1	1	-	-	-	-
Medullary	Carcinoma	3	4	-	-	-	-	-	2	-	-
	Nipple	-	2	-	-	-	-	-	-	-	23
Mucinous	Carcinoma	1	1	-	-	-	-	-	-	-	-
	Nipple	-	-	-	-	-	-	-	-	-	38
Total carcinoma (%)	Carcinoma	7 (24.1)	19 (65.5)	2 (6.9)	2	-	2	-	4 (13.8)	2	
	Nipple	6 (21)	8 (27.6)	3 (10.3)	4	4	3	2	-	-	
